# Validity and Reproducibility of a Food Frequency Questionnaire to Assess Macro and Micro-Nutrient Intake among a Convenience Cohort of Healthy Adult Qataris

**DOI:** 10.3390/nu13062002

**Published:** 2021-06-10

**Authors:** Hiba Bawadi, Rand T. Akasheh, Abdelhamid Kerkadi, Salma Haydar, Reema Tayyem, Zumin Shi

**Affiliations:** 1 Department of Nutrition, College of Health Sciences, QU-Health, Qatar University, Doha P.O. Box 2713, Qatar; abdel.hamid@qu.edu.qa (A.K.); salmahayder1@gmail.com (S.H.); reema.tayyem@qu.edu.qa (R.T.); zumin@qu.edu.qa (Z.S.); 2 Department of Nutrition and Dietetics, American University of Madaba, Madaba 11821, Jordan; r.akasheh@aum.edu.jo

**Keywords:** Qatar, FFQ, validity

## Abstract

This study aimed at developing a valid culture-sensitive quantitative food frequency questionnaire (FFQ) for Qatari adults. A convenient sample of healthy Qataris (*n* = 107) were recruited from family members of Qatar University students. The Diet History Questionnaire II of the US National Cancer Institute was translated to Arabic language, back-translated to English, pilot tested, and then modified accordingly to be used in Qatari setting. Participants were asked to complete the translated version of the FFQ. This FFQ was then validated against three 24 h diet recall (24 hDR) including a weekend day. Participants were asked to complete the FFQ again after one-month period to measure its repeatability. Dietary data were analyzed using the dietary analysis software ESHA. The validity and reliability of FFQ were assessed by comparing the median intake of nutrients and foods and by calculating the Pearson correlation coefficients. The median nutrient intakes assessed by the second FFQ were higher than that reported in the baseline FFQ1 except for fat. The percentage of increase varies between 1.5% and 96%. Results of the second FFQ indicated an overestimation of intake for most nutrients (macro and micro). Macronutrient intakes assessed by the two FFQ and 24 hDR were strongly correlated. The correlation coefficients for micronutrient intakes between FFQ2 and 24hDR were lower than that of the two FFQs except for calcium (r = 0.55) and sodium (r = 0.643). They ranged from (−0.17) for fluorine to (0.643) for sodium. The agreement rates for classifying macronutrient intakes into same or adjacent quartile were between 79.4% and 100% for the two FFQs and between 71% and 100% for the second FFQ and 24hDR. The reported consumption of food groups estimated by FFQ2 was significantly higher than that reported by FFQ1. In conclusion, the developed FFQ was sufficiently valid to assess energy and macronutrients but not micronutrients. The reliability was adequate for most nutrients.

## 1. Introduction

Different dietary assessment methods had been created to evaluate food and nutrients intake among individuals and populations. Food frequency questionnaire (FFQ) is considered an essential tool in epidemiologic research, and it is usually used to estimate the long-term relationship between diet and chronic diseases [1]. Food records and 24hDR may provide accurate figures on diet although they are costly to be used in epidemiological studies. The 24 h diet recalls (24hDR) is a retrospective method of diet assessment, where individuals are interviewed about their food and beverage consumption during the preceding 24 h [2]. However, a single 24hDR could not be considered representative of habitual diet at an individual level. At least 3-day recalls were recommended as the most appropriate method of dietary assessment [2]. Food records necessitate a high level of literacy and cooperation and at least three days would be required to assess the current intake of nutrients and foods. This makes them less practical for epidemiological studies [2]. At present, FFQ is the preferred dietary assessment method in most epidemiological studies primarily because they are somehow inexpensive and easy to administer. Hence, FFQs have been continuously modified and validated to reflect each population’s traditional foods and true food intake [3,4,5,6].

Health challenges in Qatar, including the high prevalence of obesity and diabetes, urge the need for scientific research that expose risk factors—including diet—for these chronic diseases. Despite the research boom being witnessed in Qatar and despite having obesity and diabetes control as a national priority, there is no validated dietary tool available for use in Qatari settings. Therefore, developing a FFQ specific for the Qatari population is essential. The aim of the present study was to create a culture-sensitive quantitative FFQ for Qatari adults and to validate it against three-day 24hDR collected from a sample of Qatari population.

## 2. Materials and Methods

### 2.1. Study Design and Participants

This study was approved by the IRB committee of Qatar University with an approval number of (QUSG-CAS-DHS-14\15-2). In this cross-sectional survey, a convenience sample of 500 Qatari adults from both genders were invited to participate in the study. Exclusion criteria entailed those who were suffering from chronic diseases that require dietary modifications such as diabetes, renal, and liver diseases and many others. Participants were asked to sign a consent form before completing the survey. One hundred and seven completed the study (40 males and 67 females), with an average age of 33 years

### 2.2. Dietary Assessment

The Diet History Questionnaire II of the US National Cancer Institute provides structured questions about the frequency of intake of 153 food items over a 12-month period and provides choices of three portion sizes for food quantification. This DHQ was modified according to Qatari setting to create an FFQ, which was then tested for validity and reliability. The DHQ II was first translated back by two bilingual experts. The first expert translated the first half of the DHQ into Arabic while the second translator translated the second half of the questionnaire. Thereafter, the questionnaire was back-translated to English where the first translator took the second half of the translated questionnaire and the second translator took its first half. An expert panel composed from researchers and translators were held to finalize the Arabic version of the questionnaire.

A pilot screening was conducted on 50 individuals from different geographical areas in Qatar. The original FFQ was provided and participants were asked to identify uncommon and rarely consumed foods by Qataris. Participants were also asked to provide information about food items commonly consumed by Qataris but not included in the FFQ. Based on the data collected from the pilot test, food items common in the Qatari cuisine were added to the questionnaire, while items not relevant to the Qatari’s culture were removed. In the initial visit, participants were asked to complete a paper version of the translated FFQ and were oriented how to complete the dietary recall. Participants were told to start the recall from the most recent point and recall 24 h. They were also instructed to go on 2 rounds of recalls for each day. In the first round, they will recall the major food items and in the second round, they recall the details related to amounts, preparation, toppings, etc. FFQ data collection was done via face-to-face interviews conducted by trained nutritionist to help participants complete the questionnaire. Three nutritionists were involved in data collection. To avoid variations between interviews, all nutritionists were oriented and trained of how to avoid leading questions, bias, and how to ask for additional details when needed.

Food models and standard measuring tools were used to help participants in estimating the consumed portion size. Participants’ responses were converted into average daily intakes. On the other hand, three non-consecutive 24hDR (including a weekend day) were collected from participants. The 24hDRs were gathered a week after FFQ completion in order to reduce possible intra-individual day-to-day variation. Participants were asked to complete the FFQ two times separated by a one-month period to measure its repeatability.

Food lists in the modified FFQ questions were classified based on types of foods: 21 items of vegetables; 16 items of meat such as red meat (lamb and beef), chicken, fish, cold meat, and others; 21 items of fruits and juices; nine items of milk and dairy products; eight items of cereals; four items of beans; four items of soups and sauces; five items of drinks; nine items of snacks and sweets; and 14 items of herbs and spices. The calculation of the amount of the food consumed was performed as follows: the average of the frequency category will be multiplied by the portion size then divide it by the number of days to provide the food amount/day.

Dietary data were analyzed to estimate nutrient content using the dietary analysis software (ESHA Food Processor SQL version 10.1.1; ESHA, Salem, OR, USA) with additional data on foods consumed in Qatar. The nutrients’ content of the mixed recipes and traditional foods was calculated from the Gulf Countries food composition table [7].

### 2.3. Statistical Analyses

The estimated intakes of energy, macronutrients, and micronutrients were used to assess the validity of the FFQ. Means (±SDs) as well as medians were calculated for energy and total nutrient intakes from the average of three-day 24hDRs and of each FFQ. In the study, we assessed the validity and reliability of the FFQ by (1) comparing the median intake of nutrient and food; and (2) calculating the correlations (Pearson correlation coefficients) between nutrient and food intakes derived from different dietary survey methods (FFQ vs. 24hDR) and different surveys (first and second FFQ). Wilcoxon signed rank test was also conducted to compare the nutrient intakes measured by different methods. In addition, agreement rates by quartile distribution of nutrient and food intake were also calculated.

## 3. Results

Table 1 presents the comparison of median intakes of nutrients between the baseline FFQ, second FFQ and the average of 24hDRs. The median nutrient intakes assessed by the second FFQ were higher than that reported in the baseline FFQ1, except for fat. The percentage of increase varied between 15% and 96%. Results of second FFQ indicated an overestimation of intake for most nutrients, both macros and micros. The median nutrient intakes derived from FFQ2 were higher for trans-fat, vitamin A, selenium, omega 3, and omega 6 fatty acids as compared to FFQ1. To get more details on level of validity of the FFQ, Bland-Altman plots were obtained for energy and macronutrients intake. Based on the Bland-Altman plots, the FFQ had a good agreement with food record. The Bland-Altman plots are available online as supplementary Figure S1.

Table 2 and Table 3 shows the correlations of nutrient intakes assessed by two FFQs and 24hDR along with the agreement rates by quartile distributions. Results indicated that energy and macronutrient intakes assessed by the two FFQ were strongly correlated and all correlation coefficients were above 0.5 (average r = 0.799). The same results were reported for energy and macronutrient intakes assessed by FFQ2 and 24hDR where correlation coefficients were between 0.545 (fiber) and 0.974 (energy), except for trans-fat (0.076), omega 3 (0.263), and omega 6 (0.352). These findings parallel the agreement rates between the two FFQs and 24hDR in classifying energy and macronutrients. For energy, 100% of subjects were classified in the same or adjacent quartile of intake derived from the two FFQs or FFQ2 and 24hDR. Moreover, the agreement rates for classifying macronutrient intakes into the same or adjacent quartiles were between 79.5% and 100% for the two FFQs and between 71% and 100% for second FFQ and 24hDR; the lowest being again for trans-fat. Extreme misclassification into the opposite quartile was low and ranged between 0% and 8.4% for the two FFQs and the two methods. Taken together, these findings indicated that the FFQ is valid and reliable for the estimation of energy and macronutrients.

As for micronutrient intakes, the correlation coefficients between the two FFQs were in general higher than 0.5 for all except for vitamin A (r = 0.37), vitamin B6 (r = 0.011), vitamin K (r = 0.276), chromium (r = 0.165), fluorine (r = 0.257), and iodine (r = 0.339). Furthermore, 69.2% to 98% of subjects were classified in the same or adjacent quartile of FFQs. On the other hand, the correlation coefficients for micronutrient intakes between FFQ2 and 24hDR were lower than that of two FFQs, except for calcium (r = 0.55) and sodium (r = 0.643), and ranged from (r = −0.17) for fluorine to (r = 0.643) for sodium. Moreover, 52% to 87% of participants were classified in the same or adjacent quartile intake based on FFQ and 24hDR. Extreme misclassification into the opposite quartile was between 0% to 6.5% for reliability and 1.9% to 18.5% for validity. Overall, these data indicated that the FFQ is reliable with relatively weak validity for estimating most micronutrient intakes.

Median of food intakes are presented in Table 4. The reported consumption of food groups estimated by FFQ2 was significantly higher than that reported by FFQ1, and parallels the findings on nutrient intakes. The median intakes of fruit, vegetables, grains, and milk derived from FFQ2 were significantly lower than those reported by 24hDR. No significant differences in median meat intakes between the two methods were detected.

Pearson correlation coefficients for food groups are presented in Table 5 and Table 6. Results indicate a strong correlation for all food groups between the two FFQs with an average r of 0.749, but a weak correlation for vegetable (0.028) and milk (0.06) between FFQ2 and 24hDR, and a negative correlation for fruit (−0.13), grain (−0.057), and meat (−0.192) between FFQ2 and 24hDR. When the median of food group intakes were categorized into quartiles, the ranges of agreement rates for the same or adjacent quartile were 84–95% for intakes reported by two FFQs, and 57–67% for those of 2nd FFQ and 24hDR. Classification into the opposite quartile was lower than 3% for the two FFQs and 8–16% for second FFQ and 24hDR (Table 5). In summary, we observed that the FFQ is of strong reliability but relatively weak validity for estimating food group intakes.

## 4. Discussion

Evaluating dietary habits in Qatar may help in spotting dietary risk factors that are likely contributing to the high obesity and diabetes rates in this country. Food frequency questionnaires are practical tools to assess the dietary intakes of large populations. However, FFQs have to be validated against the time consuming—yet more accurate—24hDR. The aim of this study was to develop a FFQ tailored to the people in Qatar. To our knowledge, our study is the first to attempt to create and validate a FFQ for this population. We translated the US National Cancer Institute DHQII and modified it to contain foods that are commonly consumed in Qatar. The FFQ contained questions on a total of 72 food items, administered to all participants twice, each one month apart, to assess the reliability. The average of three 24hDRs of the same participants was used to validate the FFQ. We used correlations, percentages of median difference, and agreement rates of distribution into quartiles to evaluate the validity and reliability of this FFQ. These are the standard statistical methods that have been typically and frequently used for this purpose [8,9,10,11,12,13,14,15,16,17,18,19]

Our results indicate that there was high agreement between intake of energy and macronutrient estimates from FFQ1 and FFQ2. This implies that the FFQ is reliable when estimating energy and macronutrients. When median intakes of energy, carbohydrates, protein, and fat estimated from FFQ2 and 24 h recall, results were also acceptable and reflect close estimates (Percentage of median difference ranged from 0.5% to 8%). Less valid data was obtained for micronutrients and food groups. This implies that the FFQ we developed was successful in predicting nutrient intakes, and indicates that the Qatari FFQ is valid for this purpose. Interestingly, the FFQ was remarkably successful in estimating energy intake in the study population, with a high correlation coefficient of 0.974 and a 98.13% agreement rate with the energy intake reported through the 24hDR. This correlation is much stronger than what was previously reported on FFQs created for other populations. For example, a correlation coefficient of 0.64 was reported between FFQ and 24hDR in the Shanghai’s Women Health Study, and 0.37 in a Canadian study [20,21]. Accurate estimation of energy intake through FFQ is important for drawing true conclusions on the connections between dietary habits and diseases. Similar results have also been reported in the Belgium and the Dutch studies [22,23].

On the other hand, while the reliability of our FFQ was high in estimating most nutrients, its validity—especially for micronutrients—was relatively low as evidenced by low agreement rates (i.e., 19.23% for vitamin B6 between the two methods). Correlation coefficients of nutrient intakes ranged between −0.17 (fluorine) and 0.803 (carbohydrates) for validity, and 0.011 (vitamin B6) and 0.913 (carbohydrates) for reliability. In comparison, El Kinany et al. used an adapted FFQ from the European Global Asthma and Allergy Network (GA2LEN) including traditional Moroccan foods, and found that the de-attenuated correlations for all nutrients were statistically significant and positive, ranging from 0.24 (fiber) to 0.93 (total MUFA). For reproducibility, the intra-class correlation coefficient were statistically significant and ranged between 0.69 for fat and 0.84 for Vitamin A [24]. While, in the Shanghai study, Zang et al. reported that the adjusted Spearman’s correlations were 0.33–0.77 for validity and 0.46–0.79 for reliability [8]. Moreover, in a Peruvian study, Rodriguez et al. reported high validity, with an average Pearson’s correlation coefficient of 0.70, and high reproducibility, with an average Pearson’s correlation coefficient of 0.67 [25].

In a meta-analysis of the reproducibility of food frequency questionnaires, Cui and colleagues suggested that FFQs with a minimum correlation coefficient of 0.5 for most nutrients may be considered reliable [26]. According to this criterion, we regard our FFQ to be reliable in general, with an average reliability coefficient of 0.625 for all nutrients, and higher than 0.5 for most nutrients except for vitamins A and K, zinc, iodine, chromium, and fluorine. This also paralleled good FFQ reliability of 0.749 for food group estimates. Importantly, the aforementioned meta-analysis also found that FFQ reproducibility may be improved with more food items, a 12-month interval for 24hDR—as opposed to less than 12 months—and shorter intervals between FFQs [25]. Therefore, future studies using our Qatari FFQ may improve reproducibility by modifying the dietary assessment protocol we used in our study.

Previous studies collected FFQs with a one-year interval. Since limited reproducibility of FFQ is a known limitation of this method [26], we administered the two FFQs at a one-month interval to reduce intra-individual and seasonal variations in dietary choices. However, FFQ2 tended to overestimate nutrient intakes compared to FFQ1 and 24hDR for both macronutrients and micronutrients in our study. While this may imply limited reproducibility of our FFQ, it could also reflect true changes in dietary intake or over-reporting in the FFQ2. Furthermore, FFQ2 also overestimated the intakes of some of the food groups including fruits, vegetables, grains, and dairy. Correlation coefficients of food groups ranged between 0.614 (grains) and 0.911 (fruit) for reliability, and −0.192 (meat) to 0.028 (vegetables) for validity. Likewise, the estimations of micronutrients were poorly correlated between the two methods in our study, with some estimates even being negatively correlated between FFQ and 24hDR. However, since FFQ2 estimations for energy and macronutrients remained valid and reliable, this again likely means that our FFQ2 data are presenting true changes in food group and micronutrient intake patterns. Nonetheless, the food groups’ results should be interpreted cautiously when using the FFQ.

Another possible explanation for the discrepancy in estimating micronutrients between FFQ and 24hDR is that using energy adjustment in our analysis may have led to overestimation by FFQ2. Thus, using absolute rather than relative estimates of nutrient intakes may circumvent this issue. Furthermore, while collecting additional 24hDRs around the time we conducted the FFQ2 may have provided a better explanation of this discrepancy, this may sensitize study participants to their food intake, making them answer the FFQ more accurately. The resulting training effect could then overinflate the true validity of FFQ. This issue has been previously described [21]. Additionally, it is well established that FFQ validity is greatly influenced by sex and to a lesser extent by age, BMI, and supplement use. Better validity in estimating many of the nutrients could be obtained if we adjust to remove the confounding effects of these variables [27,28].

This study has several strengths and limitations. A strength of this study was that it used primary data and used three 24 h recall. However, the sample size, as well as un ability to use biomarkers for FFQ validation. Another limitation of the study is the use of 24hDRs as the gold standard to compare with in the validation. It is known that 24hDRs does not reflect the long term intake. The use of FFQ can be reasonably reliable to rank individuals in epidemiological studies.

In conclusion, we developed a Qatari FFQ that provides a novel, comprehensive, and culturally-sensitive method for dietary evaluation that is suitable for this population. The performance of this FFQ fared better than many previously validated FFQs cited in epidemiological studies, at least for the estimation of energy and macronutrient intakes. It also performed reliably in evaluating food groups and most nutrient intakes. Future studies may focus on improving the validity of this FFQ for estimating micronutrient intakes.

## Figures and Tables

**Table 1:** Median comparison of nutrients intake between two FFQ surveys and average of the 24hDR recalls among adults.

	Median (25–75th Percentile)			Wilcoxon Signed Racked Test (*p*-Value)		Percentage of Median Difference	
Daily Intake	1st FFQ (25–75th)	2nd FFQ (25–75th)	24 h Recalls (25–75th)	1st FFQ vs. 2nd FFQ	2nd FFQ vs. 24 h Recalls	1st FFQ vs. 2nd FFQ (%)	2nd FFQ vs. 24 h Recalls (%)
Energy intake (kcal)	2092 (1663, 2277)	2123 (1669, 2576)	2132 (1544, 2714)	0.00	0.30	−1.4	−0.5
Protein (g)	79.3 (61.8, 102.1)	78.8 (66.0, 108.6)	76.1 (54.9, 100.8)	<0.01	<0.01	0.6	3.6
Carbohydrate (g)	243.1 (192.2, 299.3)	283.3 (226.0, 333.1)	264.0 (194.9, 343.0)	0.00	<0.01	−14.2	7.3
Fat (g)	81.9 (62.1, 109.2)	71.3 (55.5, 92.0)	77.6 (54.9, 113.8)	0.00	<0.01	15.0	−8.1
Saturated fat (g)	26.4 (17.3, 37.5)	24.4 (18.0, 35.0)	19.6 (13.2, 29.6)	<0.01	0.00	8.4	24.5
Monosaturated fat (g)	9.1 (4.4, 14.7)	14.2 (10.7, 20.3)	24.1 (14.1, 37.9)	0.00	0.00	−36.0	−41.3
Polysaturated fat (g)	5.5 (2.0, 7.1)	7.9 (5.8, 11.3)	17.7 (9.5, 28.1)	0.00	0.00	−30.7	−55.7
Trans fat (g)	0.33 (0.03, 0.68)	0.86 (0.38, 1.88)	0.29 (0.02, 1.04)	0.00	0.00	−61.6	196.6
Fiber (g)	17.6 (13.5, 22.5)	21.7 (16.7, 29.3)	16.9 (12.3, 25.1)	0.00	0.00	−18.6	28.3
Vitamin A (μg)	1391 (846, 2963)	6800 (3789, 10,376)	2361 (1251, 4878)	0.00	0.00	−79.5	188.0
Vitamin B1 (mg)	0.67 (0.51, 0.89)	1.09 (0.85, 1.52)	1.5 (1.2, 2.0)	0.00	0.00	−38.2	−26.2
Vitamin B2 (mg)	0.53 (0.34, 0.76)	1.11 (0.81, 1.52)	1.5 (1.1, 2.1)	0.00	0.00	−52.3	−26.7
Vitamin B3 (mg)	9.6 (5.8, 15.0)	14.5 (11.5, 20.4)	18.4 (12.9, 24.0)	0.00	<0.01	−33.7	−21.4
Vitamin B6 (mg)	0.64 (0.37, 0.84)	1.10 (0.82, 1.68)	1.35 (0.90, 2.18)	0.00	0.02	−41.6	−18.6
Vitamin C (mg)	64.5 (27.9, 116.9)	105.1 (71.1, 147.0)	86.1 (38.4, 149.5)	0.00	0.01	−38.6	22.1
Vitamin D (μg)	2.4 (0.0, 28.8)	11.3 (6.9, 23.5)	8.5 (1.1, 24.8)	0.01	0.13	−78.3	32.3
Folate	152.5 (125.5, 203.2)	269.0 (193.3, 326.2)	366.6 (269.0, 465.7)	0.00	0.00	−43.3	−26.6
Vitamin K (mcg)	10.6 (3.0, 26.3)	98.9 (40.2, 181.9)	177.3 (94.3, 323.9)	0.00	0.00	−89.3	−44.2
B5	2.0 (1.1, 2.9)	3.7 (2.8, 4.7)	4.6 (3.7, 5.7)	0.00	0.00	−45.2	−20.1
Calcium (mg)	490.6 (379.9, 732.8)	644.4 (486.3, 833.2)	583.0 (351.1, 758.0)	0.00	<0.01	−23.9	10.5
Chromium	0.89 (0.22, 1.57)	2.0 (1.4, 2.9)	2.8 (1.6, 3.8)	0.00	<0.01	−55.1	−28.8
Copper	0.44 (0.34, 0.62)	0.73 (0.57, 0.98)	0.91 (0.71, 1.22)	0.00	0.00	−39.3	−20.3
Fluorine	0.01 (0.00, 0.05)	0.18 (0.12, 0.27)	0.25 (0.18, 0.46)	0.00	<0.01	−94.3	−30.0
Iodine	6.3 (2.6, 11.8)	28.2 (18.3, 36.9)	37.0 (24.4, 53.1)	0.00	0.00	−77.6	−23.8
Iron	10.9 (8.7, 13.0)	13.0 (10.7, 16.6)	14.7 (11.6, 20.1)	0.00	<0.01	−16.5	−11.4
Phosphate	431.2 (264.4, 685.6)	668.7 (530.3, 852.0)	808.9 (578.4, 992.0)	0.00	<0.01	−35.5	−17.3
Selenium (mcg)	39.0 (21.4, 66.2)	67.4 (46.4, 85.3)	29.3 (16.2, 52.9)	0.00	0.00	−42.2	130.2
Sodium (mg)	2824 (2229, 4106)	3377 (2601, 4738)	3732 (2342, 5055)	0.00	0.19	−16.4	−9.5
Zinc (mg)	3.2 (2.3, 4.4)	5.3 (4.0, 6.9)	6.1 (4.4, 8.5)	0.00	<0.01	−39.5	−13.8
Omega 3 (g)	0.32 (0.10, 0.91)	0.56 (0.33, 0.79)	0.33 (0.18, 0.65)	0.00	<0.01	−42.3	68.2
Omega 6 (g)	2.9 (1.1, 6.2)	5.8 (4.3, 9.5)	4.5 (2.0, 6.8)	0.00	0.00	−49.9	28.9

**Table 2:** Pearson’s correlation of nutrients intake between two FFQ, 24 h recall.

Nutrients	1st FFQ vs. 2nd FFQ	2nd FFQ vs. 24 h Recalls
Energy	0.991	0.974
Protein	0.903	0.610
Carbohydrates	0.931	0.803
Fat	0.929	0.799
Saturated fat	0.856	0.670
Monosaturated fat	0.651	0.617
Polysaturated fat	0.735	0.610
Trans fat	0.553	0.076
Fiber	0.652	0.545
Vitamin A	0.370	−0.014
Vitamin B1	0.710	−0.080
Vitamin B2	0.551	−0.106
Vitamin B3	0.722	−0.080
Vitamin B6	0.011	−0.034
Vitamin C	0.677	0.176
Vitamin D	0.535	0.098
Folate	0.563	−0.135
Vitamin K	0.276	−0.054
B5	0.624	−0.105
Calcium	0.764	0.550
Chromium	0.165	−0.008
Copper	0.609	0.034
Fluorine	0.257	−0.170
Iodine	0.339	−0.138
Iron	0.822	−0.095
Phosphate	0.643	0.031
Selenium	0.760	0.116
Sodium	0.853	0.643
Zinc	0.351	0.094
Omega 3	0.840	0.263
Omega 6	0.746	0.352

**Table 3:** Agreement in quartile distribution of nutrients intake between two FFQ surveys and 24 h recall.

	Percent of Agreement							
	Same Quartile		Adjacent Quartile		Skip One Quartile		Opposite Quartile	
Daily Intake	1st FFQ vs. 2nd FFQ	2nd FFQ vs. 24 h Recalls	1st FFQ vs. 2nd FFQ	2nd FFQ vs. 24 h Recalls	1st FFQ vs. 2nd FFQ	2nd FFQ vs. 24 h Recalls	1st FFQ vs. 2nd FFQ	2nd FFQ vs. 24 h Recalls
Energy intake (kcal)	100	98.13	0	1.87	0	0	0	0
Protein (g)	71.03	46.73	28.04	42.99	0.93	6.54	0	3.74
Carbohydrate (g)	72.9	56.07	26.17	40.19	0.93	3.74	0	0
Fat (g)	75.7	62.62	24.3	30.84	0	6.54	0	0
Saturated fat (g)	68.22	39.25	29.91	50.47	1.87	9.35	0	0.93
Monosaturated fat (g)	49.53	42.06	39.25	38.32	10.28	18.69	0.93	0.93
Polysaturated fat (g)	54.21	38.32	32.71	41.12	10.28	16.82	2.8	3.74
Trans fat (g)	40.19	27.1	39.25	43.93	14.95	24.3	5.61	4.67
Fiber (g)	57.01	38.32	30.84	38.32	11.21	17.76	0.93	5.61
Vitamin A (μg)	34.58	28.97	34.58	39.25	28.04	19.63	2.8	12.15
Vitamin B1 (mg)	50.47	20.19	42.06	39.42	6.54	26.92	0.93	13.46
Vitamin B2 (mg)	37.38	26.92	46.73	31.73	14.02	25.96	1.87	15.38
Vitamin B3 (mg)	51.4	20.19	43.93	37.5	3.74	27.88	0.93	14.42
Vitamin B6 (mg)	39.25	19.23	41.12	41.35	13.08	24.04	6.54	15.38
Vitamin C (mg)	44.86	28.97	45.79	38.32	7.48	28.97	1.87	3.74
Vitamin D (μg)	54.21	22.43	36.45	44.86	8.41	23.36	0.93	9.35
Folate	42.06	25	42.99	28.85	12.15	28.85	2.8	17.31
Vitamin K (mcg)	29.91	25	42.99	35.58	20.56	24.04	6.54	15.38
B5	43.93	23.3	46.73	43.95	9.35	23.3	0	18.45
Calcium (mg)	44.86	40.19	50.47	42.99	4.67	14.02	0	2.8
Chromium	34.58	29.13	47.66	26.21	14.95	33.01	2.8	11.65
Copper	47.66	25	43.93	42.31	7.48	23.08	0.93	9.62
Fluorine	31.78	22.33	40.19	33.01	21.5	26.21	6.54	18.45
Iodine	33.98	21.15	45.63	30.77	16.5	31.73	3.88	16.35
Iron	57.01	23.81	41.12	42.86	1.87	19.05	0	14.29
Phosphate	50.47	28.85	40.19	35.58	9.35	19.23	0	16.35
Selenium (mcg)	50.47	22.43	42.99	42.06	6.54	24.3	0	11.21
Sodium (mg)	64.49	49.53	31.78	37.38	3.74	11.21	0	1.87
Zinc (mg)	48.6	25.96	42.99	39.42	6.54	22.12	1.87	12.5
Omega 3 (g)	60.75	20.56	33.64	41.12	4.67	29.91	0.93	8.41
Omega 6 (g)	48.6	34.58	42.06	42.99	7.48	15.89	1.87	6.54

**Table 4:** Median nutrients intake from the two FFQ surveys and average of the 24 h dietary recalls among adults in Qatar.

	FFQ1			FFQ2			24 h Recalls		
	Median	P25	P75	Median	P25	P75	Median	P25	P75
Fruit	0.46	0.00	1.25	1.38	1.01	1.88	2.70	1.94	4.15
Veg	1.16	0.52	1.94	1.64	1.15	2.19	2.04	1.32	2.96
Grain	4.73	3.66	6.17	6.19	4.92	7.47	8.39	6.07	10.82
Milk	0.56	0.33	1.08	0.77	0.51	1.06	0.99	0.47	1.60
Meat	4.72	2.98	6.69	4.43	3.03	6.20	4.01	2.87	5.58

**Table 5:** Agreement in quartile distribution of food groups intake between two FFQ surveys and 24 h recall.

	Percent of Agreement							
	Same Quartile		Adjacent Quartile		Skip one Quartile		Opposite Quartile	
Daily Intake	1st FFQ vs. 2nd FFQ	2nd FFQ vs. 24 h Recalls	1st FFQ vs. 2nd FFQ	2nd FFQ vs. 24 h Recalls	1st FFQ vs. 2nd FFQ	2nd FFQ vs. 24 h Recalls	1st FFQ vs. 2nd FFQ	2nd FFQ vs. 24 h Recalls
Fruit	50.5	23.1	33.6	38.5	13.1	22.1	2.8	16.4
								
Vegetable	49.5	33.7	38.3	33.7	11.2	24.0	0.9	8.7
Grain	45.8	25.2	38.3	36.9	15.0	23.3	0.9	14.6
Milk	48.6	19.2	46.7	38.5	2.8	33.7	1.9	8.7
Meat	52.3	14.1	41.1	43.5	6.5	25.9	0	16.5

**Table 6:** Pearson correlation coefficients of food groups intake between two FFQ, 24 h recall.

	FFQ1 vs. FFQ2	FFQ2 vs. FR
Fruit	0.911	−0.130
Veg	0.749	0.028
Grain	0.614	−0.057
Milk	0.669	0.060
Meat	0.804	−0.192

## Data Availability

Data is available from the PI upon request.

## Supplementary Materials

The following are available online at https://www.mdpi.com/article/10.3390/nu13062002/s1, Figure S1: Bland-Altman plot for energy, carbohydrate, protein, fat intake.
