# Supplementary figures and images for: Validity and Reproducibility of a Food Frequency Questionnaire to Assess Macro and Micro-Nutrient Intake among a Convenience Cohort of Healthy Adult Qataris
Source: Nutrients. 2021 Jun 10;13(6):2002. doi: 10.3390/nu13062002 (PMC8230372; doi:10.3390/nu13062002)

**Figure S1.** Bland-Altman plot for energy, carbohydrate, protein, fat intake.

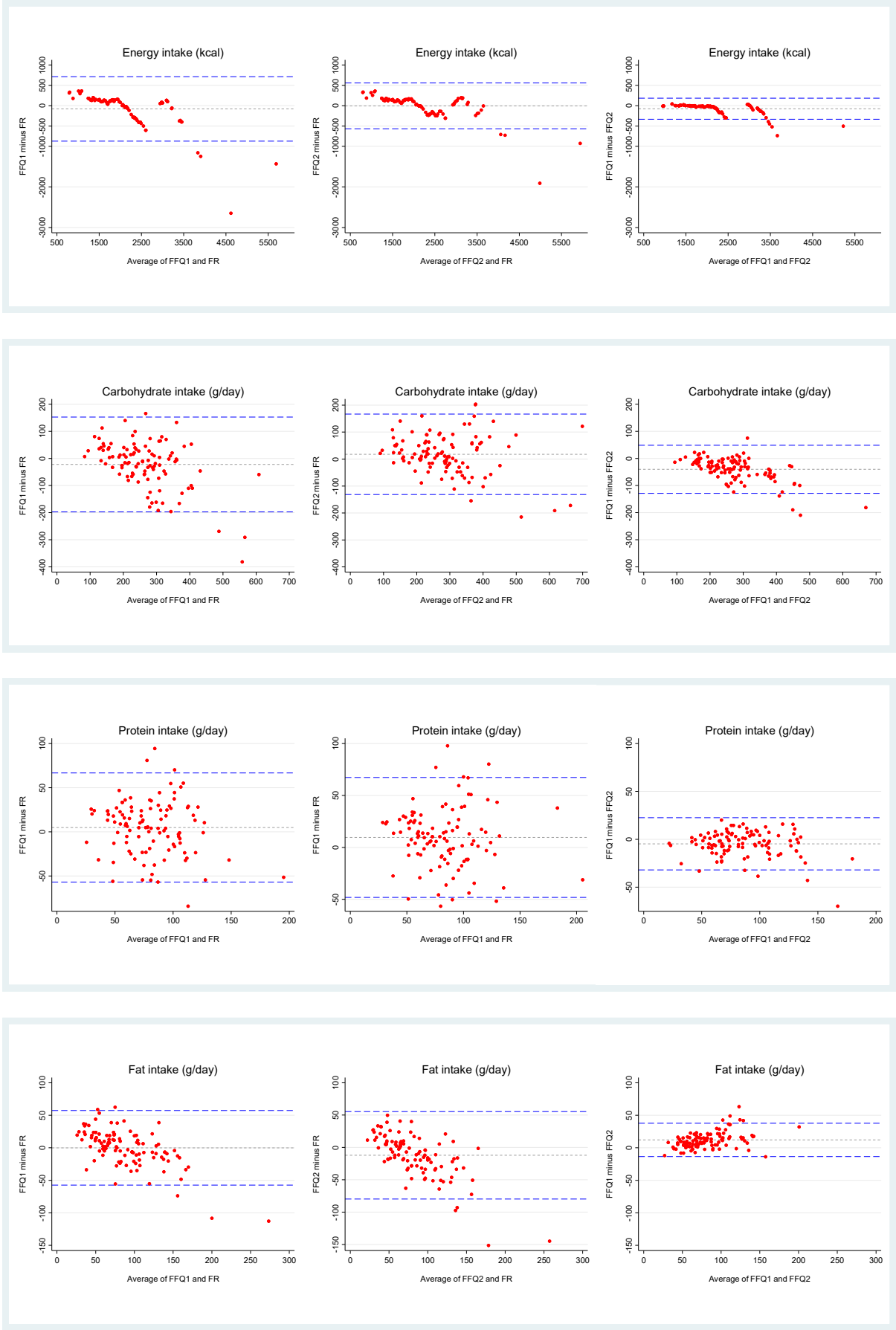

Supplement: Supplementary file 1 [file nutrients-13-02002-s001.zip › nutrients-1199222-supplementary.pdf]
